# Pain catastrophizing and pre-operative psychological state are predictive of chronic pain after joint arthroplasty of the hip, knee or shoulder: results of a prospective, comparative study at one year follow-up

**DOI:** 10.1007/s00264-022-05542-7

**Published:** 2022-08-23

**Authors:** Alexandre Hardy, Marie-Hélène Sandiford, Christophe Menigaux, Thomas Bauer, Shahnaz Klouche, Philippe Hardy

**Affiliations:** 1 Clinique du Sport, RAMSAY Santé, 36 Boulevard Saint Marcel, 75005 Paris, France; 2 Hôpital Ambroise Paré, AP-HP Université Paris Saclay, 92100 Boulogne-Billancourt, France; 3 ELSAN, 75008 Paris, France

**Keywords:** Catastrophizing, Arthroplasty, Hip, Knee, Shoulder, Psychological state

## Abstract

**Purpose:**

To assess the relationship between pre-operative psychological state, postoperative pain and function one year after total shoulder, hip or knee arthroplasty.

**Methods:**

Patients undergoing shoulder, hip or knee arthroplasty between March 2014 and November 2015 were included. Pain catastrophizing score (PCS) was determined preoperatively, at six months and at one year follow-up. Joint pain at rest was quantified using a Visual Analogue Scale (0‒100). Depressive symptoms were measured using the Beck Depression Inventory or Geriatric Depression Score, situational anxiety and dispositional anxiety were measured using the State-Trait Inventory and joint function was assessed using the Western Ontario and McMaster Universities Osteoarthritis Index for the hip and knee and Oxford Shoulder Score for the shoulder.

**Results:**

A total of 266 patients were included (65% female; mean (± SD) age: 71.8 ± 10.3 years; mean body mass index: 27.5 ± 5.0 kg/m^2^). Pre-operative PCS was significantly correlated with pain > 30/100 at one year and with functional scores, for all joints. Multivariate analysis identified the following variables as risk factors for pain intensity > 30/100 at one year: pre-operative PCS > 20/52 (OR = 3.5 ± 1.1 [95% CI: 1.9‒6.6]; *p* = 0.0001), trait-anxiety score ≥ 46 (OR = 2.4 ± 0.9 [95% CI: 1.1‒5.2]; *p* = 0.03), pre-operative pain ≥ 60/100 (OR = 3.2 ± 1 [95% CI: 1.8‒6.1]; *p* = 0.0001) and pain for at least 3 years (OR = 1.8 ± 0.6 [95% CI: 1‒3.4]; *p* = 0.04).

**Conclusion:**

Pre-operative pain catastrophizing and trait-anxiety are risk factors for post-operative pain after shoulder, hip and knee arthroplasty.

**Trial registration number:**

www.clinicaltrials.gov NCT02361359.

## Introduction

Total knee arthroplasty (TKA), total hip arthroplasty (THA) and total shoulder arthroplasty (TSA) relieve pain and improve physical performance in patients with osteoarthritis. However, 7‒23% of patients after THA and 10‒34% after TKA continue to report pain 12 months after surgery [1, 2]. After TSA, 22‒28% of patients experience persistent pain, of whom 13% have neuropathic pain [3]. Approximately 20% of patients report dissatisfaction following primary TKA [4]. However, only 5% of patients with persistent pain after TKA and 1% with persistent pain after THA have neuropathic pain [5].

One important risk factor for poor pain and functional results is pre-operative mental health, especially anxiety, affecting patients undergoing TKA [6, 7] or THA. Pain catastrophizing has been defined as an exaggerated negative orientation towards pain stimuli and pain experience that involves rumination about painful sensations, magnification of the threat of a painful stimulus and perceived inability to control pain [8]. Pre-operative catastrophizing can predict postoperative disability, number of painful body sites, reduced quality of life (QoL), negative mood and the development of chronic post-operative pain (CPOP) after various types of surgery, including THA and TKA [8–11]. However, few studies have evaluated the influence of psychological factors on shoulder arthroplasty outcomes [12–14] and, to our knowledge, the relation between catastrophizing and shoulder arthroplasty has not been investigated.

The aim of this study was to analyse the correlation between pre-operative psychological state and pain and function 1 year after TSA, THA or TKA. The secondary aim was to quantify the relationship between psychological factors and chronic joint pain at one year follow-up. Our hypothesis was that there would be a less favourable clinical outcome in patients with pre-operative psychological distress, mainly pain catastrophizing, irrespective of the joint.

## Materials and methods

### Study design and population

This prospective, single-centre study was conducted between March 2014 and November 2015. All patients undergoing TSA, THA or TKA because of primary degenerative arthrosis, > 18 years of age, autonomous and living at home were included. Exclusion criteria included the following: previous surgery on the same joint, absence of consent or patient unable to understand the questionnaires.

The study was performed in accordance with the Declaration of Helsinki and was approved by the local institutional review board (CPP). All individuals involved in the study gave their informed consent. The study was registered with https://clinicaltrials.gov/ (ClinicalTrials.gov Identifier: NCT02361359).

### Data collection and evaluation criteria

The main evaluation criterion was pain catastrophizing score (PCS) [15] determined pre-operatively, at six months and at one year of follow-up. This self-questionnaire consists of 13 items describing different ruminations and feelings that individuals may experience when they are in pain. Participants were asked to indicate the degree to which they experienced each of 13 ruminations or feelings when experiencing pain (5-point scale ranging from 0 = not at all to 4 = all the time). The PCS gives a total score and three subscale scores assessing thoughts (“I can’t stop thinking about how much it hurts”), magnification (“I worry that something serious can happen”) and helplessness (“It’s awful and I feel it overwhelms me”).

The secondary evaluation criteria were joint pain at rest quantified using a Visual Analogue Scale (VAS) from 0 (none) to 100 (worst imaginable pain), the Beck Depression Inventory (BDI) [16] or the Geriatric Depression Score (GDS) [17] to assess the presence and severity of depressive symptoms in patients > 75 years of age, the State-Trait Inventory [18] to measure situational anxiety (STAI-A) and dispositional anxiety (STAI-B), the SF12 QoL questionnaire [19], the Western Ontario and McMaster Universities Osteoarthritis Index (WOMAC) [20] for the hip and knee and the Oxford Shoulder Score (OSS) for the shoulder [21] to assess the function of the joint and the neuropathic pain questionnaire (DN4) [22]. These scores were also measured pre-operatively, after six months and at one year follow-up.

### Statistical methods

The number of patients necessary was calculated to obtain a Pearson’s correlation coefficient (*r*) of 0.8, with a confidence interval (CI) of 0.20 and a risk of error (*α*) of 0.05. The CI was 0.204 (0.676‒0.880). The sample should be at least 56 per group. Assuming a 5% rate of incomplete questionnaires, the number of patients included in each of the three groups (TSA, TKA and THA) must be at least 60, resulting in a minimum of 180 patients recruited.

For quantitative variables, differences between two independent groups were determined using Student’s *t* test, between two paired groups (before/after) using the paired Student’s *t* test and between several independent groups by analysis of variance. For qualitative variables, the chi-square test was used following a trend test in the case of a comparison of several independent groups and the McNemar test for paired groups (before/after). Correlations were investigated using Pearson’s correlation coefficient. The correlation was considered strong (*r* > 0.5), moderate (0.5 < *r* < 0.3) or weak (0.3 < *r* < 0.1). The optimal cut-off value of pre-operative PCS for pain with an intensity of > 30/100 at 1-year follow-up (yes/no) was determined by constructing receiver operating characteristic (ROC) curves, with sensitivity (Se) as the abscissa and specificity (Sp) as the ordinate. This cut-off value was chosen to obtain the highest Se and Sp possible with the best proportion of subjects well classified. The positive likelihood ratio (LR +) and negative likelihood ratio (LR −) were calculated. The diagnostic interest of preoperative PCS was evaluated from the area under the ROC curve (AUC) as follows: nil (AUC = 0.5), poorly informative (0.5 < AUC < 0.7), fairly informative (0.7 ≤ AUC < 0.9), highly informative (0.9 ≤ AUC < 1) and perfect (AUC = 1). The risk factors for pain intensity > 30/100 after 1 year were investigated by multivariate logistic regression analysis including all variables with a *p* value < 0.20 in univariate tests. A *p* value < 0.05 was considered statistically significant. All statistical analyses were carried out using Stata 10.

## Results

### Study population

**Fig. 1:**
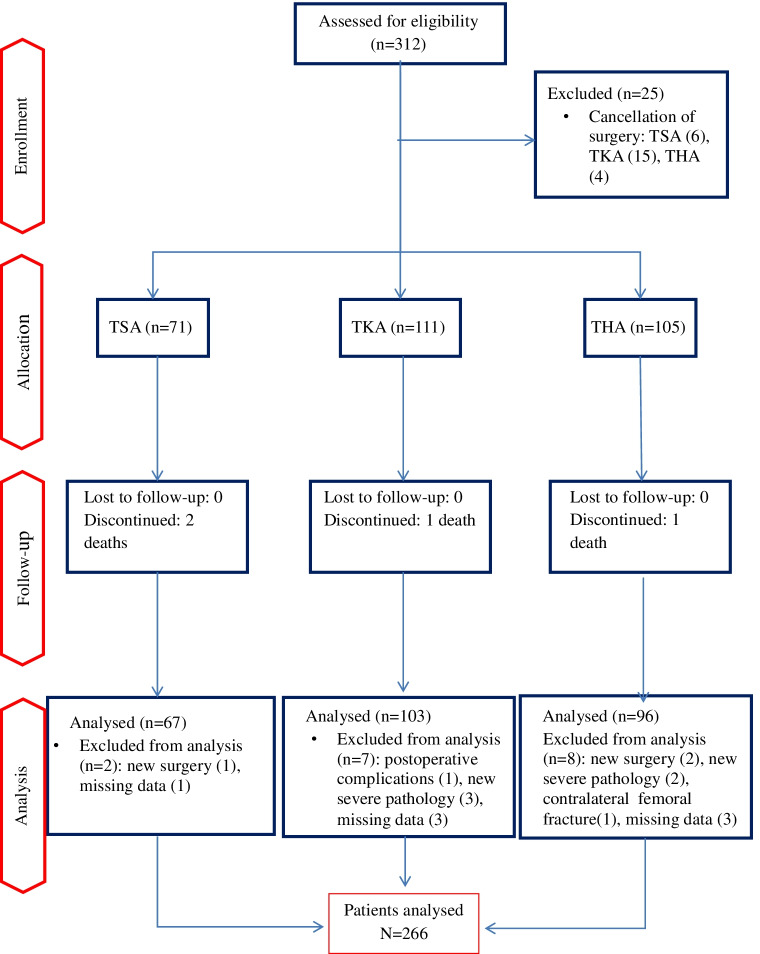
Flow chart of the study population

Three hundred and twelve patients were assessed, and 266 were included in the analysis (Fig. 1). Patients undergoing TKA were significantly younger (*p* = 0.03) and had a lower BMI (*p* = 0.004) and joint pain appeared later (*p* = 0.0002) than in the other two groups (Table 1). No significant differences in pre-operative psychological factors were observed between the three groups of patients (Table 1).

**Table 1:** Demographic characteristics and pre-operative data for the study population

Pre-operative data	All (*N* = 266)	TKA (*N* = 103)	THA (*N* = 96)	TSA (*N* = 67)	*p*
Age (years)	71.8 ± 10.3 (29‒92)	73.3 ± 9.3 (29‒92)	69.7 ± 12 (30‒92)	72.7 ± 8.9 (47‒90)	0.03
Sex, F/M	173/93 (65/35%)	67/36 (65/35%	62/34 (64.6/35.4%)	44/23 (65.7/34%)	0.99
BMI	27.5 ± 5 (15.4‒47.3)	28.7 ± 5.2 (20‒41.6)	26.4 ± 4.5 (15.4‒40.1)	27.2 ± 5.1 (16.8‒47.3)	0.004
ASA score					
1	40 (15%)	11 (10.7%)	22 (22.9%)	7 (10.5%)	0.11
2	190 (71.4%)	78 (75.7%)	61 (63.5%)	51 (76.1%)	
3	36 (13.6%)	14 (13.6%)	13 (13.6%)	9 (13.4%)	
Pre-operative VAS (/100)	56.9 ± 17.8 (0‒100)	54.5 ± 16.3 (15‒99)	57.2 ± 17.1 (25‒95)	60.1 ± 20.6 (0‒100)	0.13
Duration of pain before surgery (years)	2.4 ± 0.7 (0‒3)	2.6 ± 0.6 (0‒3)	2.2 ± 0.7 (1‒3)	2.4 ± 0.8 (1‒3)	0.0002
SF12 MS (/100)	44.8 ± 11 (19.7‒69.9)	45.3 ± 11 (23.1‒69.9)	43.2 ± 11 (19.7‒69.2)	46.2 ± 10.8 (24.9‒66.4)	0.19
SF12 PS (/100)	36.7 ± 9 (14.9‒60.1)	36.9 ± 8.9 (18.1‒60.1)	35.4 ± 9 (19.3‒57.2)	38.3 ± 9 (14.9‒54.1)	0.13
Catastrophizing (/52)	14 ± 9.5 (0‒39)	13.9 ± 9.9 (0‒39)	14.3 ± 9.1 (1‒38)	13.8 ± 9.5 (1‒36)	0.19
Helplessness (/24)	6.5 ± 4.8 (0‒18)	6.4 ± 4.6 (0‒18)	6.6 ± 4.7 (0‒18)	6.4 ± 4.5 (0‒18)	0.93
Magnification (/12)	2.8 ± 2.6 (0‒12)	2.5 ± 2.5 (0‒12)	3.1 ± 2.6 (0‒12)	2.7 ± 2.9 (0‒12)	0.23
Rumination (/16)	4.8 ± 3.6 (0‒12)	5 ± 3.8 (0‒12)	4.6 ± 3.4 (0‒12)	4.8 ± 3.5 (0‒12)	0.75
STAI trait (/80)	35.9 ± 9.8 (20‒73)	34.8 ± 9.5 (20‒58)	37 ± 10.6 (21‒73)	35.9 ± 8.8 (20‒55)	0.29
STAI trait by subgroups					
1^a^	223 (83.8%)	90 (87.4%)	75 (78.1%)	58 (86.6%)	0.15
2	33 (12.4%)	9 (8.7%)	15 (15.6%)	9 (13.4%)	
3	10 (3.8%)	4 (3.9%)	6 (6.3%)	0	
STAI state (/80)	35.7 ± 11.3 (20‒68)	33.6 ± 10 (20‒60)	37.1 ± 12.3 (20‒68)	36.9 ± 11.4 (20‒63)	0.06
STAI state by subgroups					
1^a^	214 (80.4%)	88 (85.4%)	77 (80.2%)	49 (73.1%)	0.19
2	34 (12.8%)	11 (10.7%)	10 (10.4%)	13 (19.4%)	
3	18 (6.8%)	4 (3.9%)	9 (9.4%)	5 (7.5%)	
BDI score^b^ (/39)	4.1 ± 4.7 (0‒27)	3.3 ± 3.9 (0‒18)	5 ± 5.3 (0‒27)	3.8 ± 4.2 (0‒18)	0.11
GDS score^c^ (/15)	3.7 ± 3.2 (0‒15)	3.6 ± 3.2 (0‒14)	4 ± 3.1 (0‒15)	3.6 ± 3.2 (0‒15)	0.83
Depression^d^	47 (17.7%)	15 (14.6%)	19 (19.8%)	13 (19.4%)	0.57
Neuropathic pain^e^	93 (35%)	33 (32%)	41 (42.7%)	19 (28.4%)	0.12

Data shown are mean (± standard deviation (SD)), range (min‒max) or *n* (%)

*TKA* total knee arthroplasty, *THA* total hip arthroplasty, *TSA* total shoulder arthroplasty, *F* female, *M* male, *BMI* body mass index, *VAS* Visual Analogue Scale, *ASA* American Society of Anaesthesiologists, *SF* short form, *MS* mental state, *PS* physical state, *STAI* State-Trait Inventory

^a^Weak or very weak (< 45), average (46–55), high or very high (> 56)

^b^Beck Depression Inventory < 75 years old (149 patients)

^c^Geriatric Depression Scale 15 > 75 years old (117 patients)

^d^Average to severe depression (BDI score > 8 or GDS > 7)

^e^DN4 score < 4/10

### Primary endpoint

At one year follow-up, there was no significant difference in mean (SD) pain intensity (/100) between the three groups (27 ± 20.1 TKA, 20.1 ± 19.1 THA, 23.9 ± 21.2 TSA; *p* = 0.05). However, at one year follow-up, significantly less patients undergoing TSA had neuropathic pain compared to the TKA and THA groups (9% vs. 23.7% and 27.4%, respectively; *p* = 0.01). No significant differences were found in the proportion of patients with pain intensity > 30/100 at 1 year (36.9% TKA, 22.9% THA, 34.3% TSA; *p* = 0.08). Total mean (± SD) pre-operative PCS (/52) was 13.9 ± 9.9 for TKA patients, 14.3 ± 9.1 for THA and 13.8 ± 9.5 for TSA (Table 2). Post-operative pain intensity at one year was significantly correlated with pre-operative PCS for all patients (Table 2). In TKA or THA patients, post-operative pain intensity at one year was significantly correlated with different PCS sub-items.

**Table 2:** Correlation coefficients between mean post-operative pain intensity at 1 year and pre-operative pain catastrophizing score (PCS) for all patients and by subgroup

Correlation coefficient	Pre-operative pain catastrophizing score (PCS)			
	Global (/52)	Helplessness (/24)	Amplification (/12)	Rumination (/16)
All patients				
Pain intensity, mean ± SD	14 ± 9.5	6.5 ± 4.8	2.8 ± 2.6	4.8 ± 3.6
* r*, mean	0.42	0.39	0.33	0.34
* p*	0.00001	0.00001	0.00001	0.00001
TKA patients				
Pain intensity, mean ± SD	13.9 ± 9.9	6.4 ± 4.6	2.5 ± 2.5	5 ± 3.8
* r*, mean	0.48	0.46	0.44	0.39
* p*	0.00001	0.00001	0.00001	0.00001
THA patients				
Pain intensity, mean ± SD	14.3 ± 9.1	6.6 ± 4.7	3.1 ± 2.6	4.6 ± 3.4
* r*, mean	0.50	0.49	0.32	0.41
* p*	0.00001	0.00001	0.001	0.00001
TSA patients				
Pain intensity, mean ± SD	13.8 ± 9.5	6.7 ± 5.3	2.7 ± 2.9	4.8 ± 3.5
* r*, mean	0.28 (weak)	0.20 (not significant)	0.27 (weak)	0.16 (not significant)
* p*	0.02	0.09	0.03	0.18

*TKA* total knee arthroplasty, *THA* total hip arthroplasty, *TSA* total shoulder arthroplasty, *SD* standard deviation

In TSA patients, the intensity of post-operative pain at one year was significantly but weakly correlated with pre-operative PCS, but not with the sub-items ‘helplessness’ and ‘rumination’ of catastrophism (Table 2).

### Correlation between pre-operative PCS and functional score at one year

Pre-operative PCS and functional WOMAC score at three year were significantly correlated in the TKA and THA groups (Table 3). There was a weakly significant correlation between pre-operative PCS and functional OSS score in the TSA subgroup (Table 3).

**Table 3:** Correlation coefficients between pre-operative PCS and WOMAC index at 1 year, between pre-operative PCS and functional OSS at 1 year in the TSA subgroup and univariate analysis of risk factors for pain intensity > 30/100 at 1-year follow-up

Correlation coefficient	WOMAC total (/100)	WOMAC pain (/100)	WOMAC handicap (/100)
TKA patients			
PCS, mean ± SD (range (min–max))	29.3 ± 19.6 (0‒97.9)	27 ± 20.1 (0‒95)	31 ± 20.6 (0‒100)
* r*, mean	0.48	0.48	0.44
* p*	0.00001	0.00001	0.00001
THA patients			
PCS, mean ± SD (range (min–max))	25.4 ± 19.5 (0‒75)	20.1 ± 19.1 (0‒75)	28.7 ± 21.6 (0‒78.6)
* r*, mean	0.51 (high)	0.50 (moderate)	0.51 (high)
* p*	0.00001	0.00001	0.00001
Correlation coefficient	**OSS total (/100)**	**OSS pain (/100)**	**OSS handicap (/100)**
TSA subgroup			
PCS, mean ± SD (range (min–max))	23 ± 19.4 (0‒88.6)	23.9 ± 21.2 (0‒100)	22.6 ± 22.5 (0‒100)
* r*, mean	0.29 (weak)	0.28 (weak)	0.23 (not significant)
* p*	0.02	0.02	0.07
Univariate analysis of risk factors for pain	**Pain > 30/100 (*N* = 83)**	**Pain ≤ 30/100 (*N* = 183)**	***p***
Age (years)	71.3 ± 10.2 (30‒87)	72.1 ± 10.4 (29‒92)	0.57
Sex (F/M)	59/24 (71.1/28.9%)	114/69 (62.3/37.7%)	0.16
BMI	28.2 ± 5.1 (16.9‒41.6)	27.2 ± 4.9 (15.4‒47.3)	0.14
ASA			
1	10 (12%)	30 (16.4%)	
2	60 (72.3%)	130 (71%)	
3	13 (15.7%)	23 (12.6%)	0.61
Type of prosthesis			
TKA	38 (45.8%)	65 (35.5%)	
THA	22 (26.5%)	74 (40.4%)	0.08
TSA	23 (27.7%)	44 (24.1%)	
Pre-operative VAS (/100)	64.8 ± 14.4 (25‒100)	53.3 ± 18.1 (0‒95)	0.00001
Duration of pre-operative pain (years)	2.5 ± 0.7 (1‒3)	2.3 ± 0.7 (0‒3)	0.04
Pre-operative SF12 MS (/100)	39.3 ± 10.2 (19.7‒60.2)	47.2 ± 10.4 (23.1‒69.9)	0.00001
Pre-operative SF12 PS (/100)	37.2 ± 8.3 (20.8‒55.7)	36.5 ± 9.3 (14.9‒60.1)	0.56
Pre-operative catastrophizing (/52)	20.1 ± 8.6 (1‒39)	11.3 ± 8.5 (0‒36)	0.00001
Pre-operative STAI trait (/80)	39.6 ± 9.5 (22‒62)	34.2 ± 9.4 (20‒73)	0.00001
Pre-operative STAI state (/80)	39.3 ± 11.8 (20‒68)	34.1 ± 10.7 (20‒68)	0.0005
Pre-operative depression (moderate to severe)	23 (27.7%)	24 (13.1%)	0.004
Neuropathic pain	38 (45.8%)	55 (30.1%)	0.01

*TKA* total knee arthroplasty, *THA* total hip arthroplasty, *TSA* total shoulder arthroplasty, *PCS* pain catastrophizing score, *WOMAC* Western Ontario and McMaster Universities Osteoarthritis Index, *OSS* Oxford Shoulder Score, *F* female, *M* male, *BMI* body mass index, *VAS* visual analogue scale, *ASA* American Society of Anaesthesiologists, *SF* short form, *MS* mental state, *PS* physical state, *STAI* State-Trait Inventory

In TKA or THA patients, preoperative PCS was significantly correlated with functional WOMAC score at 1 year and with WOMAC subscores ‘pain’ and ‘handicap’ (Table 3). In TSA patients, preoperative PCS was significantly but weakly correlated with functional OSS score at 1 year and was not correlated with the sub-item ‘handicap’ (Table 3).

### Pre-operative PCS cut-off values to predict pain > 30/100 at one year

#### All patients

Pre-operative PCS was fairly informative of pain > 30/100 at one year (AUC = 0.77 ± 0.03 [95% CI: 0.71‒0.83]). The optimal pre-operative PCS cut-off whatever the prosthetic joint was 20/52 (Se = 54.2%, Sp = 82.5%, patients classed correctly = 73.7%, LR +  = 3.1 and LR −  = 0.5).

#### TKA patients

In the TKA group, pre-operative PCS was fairly informative of pain intensity > 30/100 at 1 year (AUC = 0.83 ± 0.04 [95% CI: 0.75‒0.91]). The optimal pre-operative PCS cut-off was 18/52 (Se = 68.4%, Sp = 83.1%, patients classed correctly = 77.7%, LR +  = 4 and LR −  = 0.3).

#### THA patients

In the THA group, pre-operative PCS was fairly informative of pain intensity > 30/100 at 1 year (AUC = 0.81 ± 0.04 [95% CI: 0.72‒0.89]). The optimal pre-operative PCS cut-off was 19/52 (Se = 72.7%, Sp = 78.4%, patients classed correctly = 77.1%, LR +  = 3.4 and LR −  = 0.3).

#### TSA patients

In the TSA subgroup of patients, pre-operative PCS was poorly informative of pain intensity > 30/100 at 1-year (AUC = 0.67 ± 0.07 [95% CI: 0.53‒0.81]). The optimal pre-operative PCS cut-off was 23/52 (Se = 34.8%, Sp = 88.6%, patients classed correctly = 70.2%, LR +  = 3.1 and LR −  = 0.7).

### What are the risk factors for pain intensity > 30/100 at 1 year?

In univariate analysis, patients with pain intensity > 30/100 at 1 year had significantly more pre-operative pain (VAS (/100): 64.8 ± 14.4 vs. 53.3 ± 18.1 (≤ 30/100); *p* < 0.00001), had suffered from pain for longer before surgery (2.5 ± 0.7 years vs. 2.3 ± 0.7 years; *p* = 0.04), had a significantly worse QoL mentally (SF12 MS (/100): 39.3 ± 10.2 vs. 47.2 ± 10.4; *p* < 0.00001), had significantly greater pre-operative catastrophism ((/52): 20.1 ± 8.6 vs. 11.3 ± 8.5; *p* < 0.00001), were significantly more anxious (STAI trait (/80): 39.6 ± 9.5 vs. 34.2 ± 9.4; *p* < 0.00001; STAI state (/80): 39.3 ± 11.8 vs. 34.1 ± 10.7; *p* < 0.0005), were more likely to have moderate to severe depression (27.7% vs. 13.1%; *p* = 0.004) and were more likely to experience neuropathic pain (45.8% vs. 30.1%; *p* < 0.01).

Multivariate analysis included the following variables: sex, BMI (≥ 30), type of prosthesis (THA, TKA, TSA) and pre-operative pain intensity (≥ 60/100), pain duration (≥ 3 years), SF12 mental dimension (< 50%), PCS (> 20), STAI trait (≥ 46) and state (≥ 46) and existence of moderate‒severe neuropathic pain. The following were identified as risk factors for pain intensity > 30/100 at 1 year (Table 3): pre-operative PCS > 20/52 (OR = 3.5 ± 1.1 [95% CI: 1.9‒6.6], *p* = 0.0001), trait-anxiety score ≥ 46 (OR = 2.4 ± 0.9 [95% CI: 1.1‒5.2], *p* = 0.03), pre-operative pain ≥ 60/100 (OR = 3.2 ± 1 [95% CI: 1.8‒6.1], *p* = 0.0001) and pain for at least 3 years (OR = 1.8 ± 0.6 [95% CI: 1‒3.4], *p* = 0.04). After adjustment, the variable ‘type of prosthesis’ was eliminated from the final model.

### Evolution of the different psychological and functional scores between inclusion and one year follow-up

At one year follow-up, patients expressed significantly less catastrophism and were less anxious compared to their preoperative state. However, no improvement was noted in the existence of moderate‒severe depression. A significant improvement was observed in joint pain, neuropathic pain and QoL. Mean intensity of joint pain at one year (/100) was 23.7 ± 20.2 vs. 56.9 ± 17.8 pre-operatively (*p* < 10^−5^), 83 patients (31.2%) had pain intensity > 30/100 vs. 242 (91%) pre-operatively (*p* < 10^−5^) and 56 (21.1%) patients suffered from neuropathic pain vs. 90 (35%) pre-operatively (*p* = 0.0003). Function was significantly improved for all types of joint at one year (for WOMAC and OSS scores, *p* < 0.00001).

## Discussion

This study shows that pre-operative pain catastrophizing and trait-anxiety are risk factors for CPOP after THA and TKA, although catastrophizing was less strongly correlated after TSA. These results support a growing body of evidence indicating that psychological factors are prognostic for pain severity and physical function post-arthroplasty, especially after TKA [23].

Khan et al. [9] stated: “High pain catastrophizing levels after knee surgery in osteoarthritis patients have been reported to be associated with high levels of pain and disability up to six months post-operatively”, and Birch et al. [24] observed that patients with high pre-operative PCS have lower knee function and QoL and more pain four months and 12 months after knee arthroplasty. Riddle et al. [10] investigated the influence of pre-operative PCS on the WOMAC score after TKA. In their study, PCS was the predominant predictor of pain outcome. Patients with PCS > 16 had a 2.67-times increased risk of poor outcome (< 50% improvement) compared to patients with PCS ≤ 15 [10]. In a systemic review, Niederstrasser and Cook [25] highlighted the importance of patients’ psychological profiles in terms of depression, anxiety and pain catastrophizing on pain intensity and function. Lewis et al. [26] stated that pain catastrophizing was one of the strongest independent predictors of persistent pain after TKA.

We found an optimum PCS cut-off value of 18/52 for TKA, 19/52 for THA and 23/52 for TSA. Global cut-off value was 20. In contrast to the literature, we calculated the PCS cut-off value from a ROC curve. Other studies used the top tertile of the scores obtained [10]; patients with PCS > 16 were considered as “at risk” (in a dichotomous way, as in this study). In other studies, PCS was considered in a continuous way [8]. Some authors also considered PCS with its subscores ‘helplessness’, ‘magnification’ and ‘rumination’ [27]. In the latter study, mean PCS was 19.4 and mean PCS subscale scores were 3.4/11 (magnification), 7.2/16 (rumination) and 8.8/24 (helplessness).

In our study, mean PCS was 13.9 for TKA (2.5/11 magnification, 5.0/16 rumination, 6.4/24 helplessness), 14.3 for THA (3.1/11 magnification, 4.6/16 rumination, 6.6/24 helplessness) and 13.8 for TSA (2.7/11 magnification, 4.8/16 rumination, 6.7/24 helplessness).

This study has several strengths. It is the first to compare the influence of psychological factors, particularly pain catastrophizing and anxiety, on knee, hip and shoulder arthroplasty outcomes. In previous studies, pain catastrophizing was related to anxiety and depression and had an influence on TKA and THA outcomes. Our study is the first to include shoulder arthroplasty in this correlation. However, our study is limited by the number of patients, which was too small to determine the influence of depression on arthroplasty results. Depression is considered to be partly responsible for poor pain and functional results.

According to our results, catastrophizing is strongly related to CPOP after THA and TKA, but less so after TSA. This could be because the hip and knee are joints of the lower limbs and are therefore more handicapping than those of the upper limbs.

A recent publication [23] showed that generalised anxiety disorder could be a modifiable risk factor for pain catastrophizing, strengthening the interest of publishing this study, even six years after the one year follow-up. Indeed, our results suggest that the pre-operative administration of self-questionnaires may help to identify a group of patients at “high-risk” of persistent pain after THA, TKA or TSA. Theunissen et al. [11] suggested that anxiety should be investigated to prevent CPOP. Pain catastrophizing should also be checked routinely before surgery. The pre-operative identification of patients with pain catastrophizing may enable short psychological interventions, including relaxation and/or breathing techniques and cognitive behavioural therapy, to help change and adapt catastrophizing beliefs [9, 10]. Riddle et al. [10] conducted a study in patients undergoing TKA to develop their pain coping skills. The intervention was provided by eight telephone-based sessions around the time of surgery, to teach the patients to identify irrational, maladaptive thoughts and to replace these with alternative, rational coping thoughts. Keogh et al. [28] conducted a study in which the intervention of a psychologist improved the PCS by up to 40%. These therapies may facilitate a reduction in catastrophic thinking, with the goal of reducing post-operative pain and improving joint function.

In conclusion, high pre-operative catastrophizing and trait-anxiety are risk factors for CPOP after shoulder, hip and knee arthroplasty.

## Abbreviations

BDI: Beck Depression Inventory
CPOP: Chronic postoperative pain
GDS: Geriatric depression score
MS: Mental state
OSS: Oxford Shoulder Score
PCS: Pain catastrophizing score
PS: Physical state
QoL: Quality of life
STAI-A: State-Trait Inventory to measure situational anxiety
STAI-B: State-Trait Inventory to measure dispositional anxiety
THA: Total hip arthroplasty
TKA: Total knee arthroplasty
TSA: Total shoulder arthroplasty
VAS: Visual Analogue Scale
WOMAC: Western Ontario and McMaster Universities Osteoarthritis Index
